# Cowpox Virus Outbreak in Banded Mongooses (*Mungos mungo*) and Jaguarundis (*Herpailurus yagouaroundi*) with a Time-Delayed Infection to Humans

**DOI:** 10.1371/journal.pone.0006883

**Published:** 2009-09-03

**Authors:** Andreas Kurth, Martin Straube, Annette Kuczka, Anton Josef Dunsche, Hermann Meyer, Andreas Nitsche

**Affiliations:** 1 German Consultant Laboratory for Poxviruses, Robert Koch Institute, Berlin, Germany; 2 Zoo Krefeld, Krefeld, Germany; 3 Chemisches- und Veterinäruntersuchungsamt Rhein-Ruhr-Wupper, Krefeld, Germany; 4 Städtisches Klinikum Karlsruhe, Karlsruhe, Germany; 5 Bundeswehr Institute of Microbiology, Munich, Germany; University of California San Diego, United States of America

## Abstract

**Background:**

Often described as an extremely rare zoonosis, cowpox virus (CPXV) infections are on the increase in Germany. CPXV is rodent-borne with a broad host range and contains the largest and most complete genome of all poxviruses, including parts with high homology to variola virus (smallpox). So far, most CPXV cases have occurred individually in unvaccinated animals and humans and were caused by genetically distinguishable virus strains.

**Methodology/Principal Findings:**

Generalized CPXV infections in banded mongooses (*Mungos mungo*) and jaguarundis (*Herpailurus yagouaroundi*) at a Zoological Garden were observed with a prevalence of the affected animal group of 100% and a mortality of 30%. A subsequent serological investigation of other exotic animal species provided evidence of subclinical cases before the onset of the outbreak. Moreover, a time-delayed human cowpox virus infection caused by the identical virus strain occurred in a different geographical area indicating that handling/feeding food rats might be the common source of infection.

**Conclusions/Significance:**

Reports on the increased zoonotic transmission of orthopoxviruses have renewed interest in understanding interactions between these viruses and their hosts. The list of animals known to be susceptible to CPXV is still growing. Thus, the likely existence of unknown CPXV hosts and their distribution may present a risk for other exotic animals but also for the general public, as was shown in this outbreak. Animal breeders and suppliers of food rats represent potential multipliers and distributors of CPXV, in the context of increasingly pan-European trading. Taking the cessation of vaccination against smallpox into account, this situation contributes to the increased incidence of CPXV infections in man, particularly in younger age groups, with more complicated courses of clinical infections.

## Introduction

Attention was first drawn to poxviruses infecting exotic zoo animals in 1960, when, still in the era of smallpox vaccination, two captive Asian elephants died at the Zoological Garden in Leipzig/Germany [1]. At that time, the causative agent was believed to be vaccinia virus (VACV) that was most probably transmitted by recently vaccinated children to the elephants. However, this hypothesis was never proven. The fact that mandatory smallpox vaccination was abolished in Europe in 1980 with poxvirus outbreaks still occurring in Continental European and British zoos and circuses argues against VACV as their causative agent. To date, more than 30 outbreaks have been reported, affecting various species (Table 1). Virus isolates obtained from these outbreaks have been retrospectively characterized as cowpox virus (CPXV). CPXV belong to the genus *Orthopoxvirus* (OPV) of the family *Poxviridae*. Virions are brick-shaped with a size of around 200 nm in diameter and 350 nm in length and carries its genome of approx. 230 kbp in a single, linear, double-stranded segment of DNA [2]. Several often fatal infections among zoo and circus elephants have been reported mainly from Germany (Table 1). As a consequence, elephants are routinely vaccinated with the attenuated modified vaccinia virus Ankara (MVA) strain of vaccinia virus [3], [4]. For other exotic zoo animals, very little is known about successful vaccination and immune response to a vaccinia cowpox infection.

**Table 1:** CPXV infected exotic animals (except *Muroidae*).

Species	Geographic origin (outbreaks)	No of animals with clinical signs	No of fatal cases	Year	Reference
Asian elephant (*Elephas maximus*)	Germany (18)	>45	>8	1960–2007	[4], [8], [28]
	Austria	1	0	1974	[29]
	France	nk	nk	nk	Essbauer unpublished 2007
	The Netherlands	nk	nk	1973	[28]
	Poland	nk	nk	1977	[28]
	Czech Republic	nk	nk	1972	[28]
African elephant (*Loxodonta africana*)	Germany (7)	>15	2	1960–90	[28]
Lion (*Panthera leo*)	Russia	3	3	1973	[5]
Black panther (*Panthera padus*)	Russia	1	1	1973	[5]
Cheetah (*Acinonyx jubatus*)	Russia	2	2	1973	[5]
	England	3	2	1977	[19]
	England	3	2	1978	[19]
Puma (*Felis concolor*)	Russia	5	3	1973/74	[5]
Jaguar (*Felis onca*)	Russia	2	0	1973	[5]
Ocelot (*Felis pardalis*)	Russia	2	1	1973	[5]
Far eastern cat (*Felis bengalis*)	Russia	nk	Eutha-nized	1974	[5]
Okapi (*Okapia johnstoni*)	Denmark	2	1	1963	[30]
	The Netherlands	5	1	1968	[31]
Anteater (*Myrmecophaga tridactyla*)	Russia	2	2	1973	[5]
Black rhinoceros (*Diceros bicornis*)	Germany	2	1	1977, 2004	[28], [32]
White rhinoceros (*Ceratotherium s. simum*)	Germany	2	0	1977	[28]
Llama (*Lama glama pacos*)	Germany	7	5	1994	[33]
Patagonian cavy (*Dolichotis patagonum*)	The Netherlands	5	5	2006	[34]
	Germany	6	6	2007	Nitsche unpublished 2007
Red panda (*Ailurus fulgens*)	Germany	2	2	1997	[18]
Beaver (*Castor fibor canadensis*)	Germany	10	10	1997	[18]
Macaques (*Macaca spec.*)	The Netherlands	3	3	2003	[14]
Cebid monkeys	Germany	nk	30	2002	[35]

nk: not known.

The most dramatic outbreaks in exotic animals known so far occurred in the Moscow Zoo in 1973 and 1974, causing serious illness in six different species of the family *Felidae* [5]. Virus was recovered from 18/19 animals examined. Based on large intracellular eosinophilic A-type inclusion bodies and the appearance of hemorrhagic pocks on the chorioallantoic membrane of embryonated hen's eggs, it was characterized as CPXV. The origin of this virus appears to have been epizootics of poxvirus infections in colonies of white rats which were used as food for the carnivores [6]. In those epizootics a case-fatality rate exceeding 30% could be observed. It was assumed that the white rats were infected by accidental contact with wild Norwegian rats (*Rattus norvegicus*). Experiments performed by Maiboroda demonstrated that Norwegian rats could be productively infected with CPXV and could shed significant amounts of virus, especially if under stress [7]. A potential role of rats as part of the chain of transmission has been emphasized from an outbreak in a circus in Northern Germany, where all virus isolates obtained from asymptomatic rats, the deceased elephant and the locally infected animal care taker had an identical sequence of the hemagglutinin gene [8].

CPXV occurs naturally in several species of rodents in Europe and Western parts of Russia [2]. Although serological surveys demonstrated a high proportion of seropositive bank voles (*Clethrionomys glareolus*), field voles (*Microtus agrestis*) and wood mice (*Apodemus sylvaticus*) [9]–[12], no virus isolate has been obtained from these species so far. Experimental CPXV infection in bank voles, one of the main reservoir hosts, yielded only low infectious titers of CPXV [13]. This points to a co-evolution of virus and host over years. On the other hand, the fatal outcome in large felids, elephants and other exotic species indicates that these species are highly susceptible hosts.

In this respect, the role of rats has not yet been elucidated. Wild rats could be either a primary reservoir or an amplifying host. Little is also known about the origin of CPXV outbreaks. In very few cases of outbreaks occurring among exotic animals only rats could be identified as a potential origin [8], [14], [15]. Since other rodents were never found to be CPXV positive, both wild-living rodents and those bred as food for carnivores have to be considered as the most likely source of transmitting a CPXV infection to exotic animals.

Direct human-to-human transmission of CPXV has not been reported so far. Among other highly susceptible hosts like exotic animals an intra-species transmission could be observed repeatedly with similar clinical symptoms, indicating different virus susceptibilities among vertebrates that possibly depend on the CPXV strain. Nevertheless, despite the wide host range of CPXV, the same CPXV strain rarely infects different animal species. Likewise, conclusive evidence for the co-circulation of different CPXV strains within the same geographic region has rarely been provided but was rather assumed [8], [16]. Almost 50 years after CPXV was first detected in a species other than cattle, new CPXV hosts are still being reported, and serologic studies have determined further potentially susceptible wild and exotic animals. Although taxonomically all virus isolates have been classified as cowpox, the terms elephantpox, catpox and ratpox are used simultaneously in the scientific community as synonyms, depending on the species from which the respective virus was isolated. The clinical picture of CPXV infection in different animals is rather similar regardless of the infected species and mostly results in cutaneous lesions. Less often there are pulmonary symptoms without skin lesions. CPXV infections are epitheliotropic, often starting as vesicular lesions, then developing into a pustule with an indented centre and a raised erythematous border. The mortality among exotic animals and felids is high, although exact data are lacking. In humans CPXV infections usually remain localized and are self-limiting but can become fatal in immunosuppressed patients [17].

This is the first description of a generalized CPXV infection in banded mongooses (*Mungos mungo*) and jaguarundis (*Herpailurus yagouaroundi*) which occurred at the Zoological Garden in Krefeld in the spring of 2008. A subsequent serological investigation of other exotic animal species living in this zoo provides evidence for subclinical infection before the onset of clinical cases in the mongoose colony. Moreover, this is the first report of a time-delayed CPXV infection with an identical virus strain occurring in different geographical areas, indicating a common source of infection.

## Results

### The outbreak

**Figure 1:**
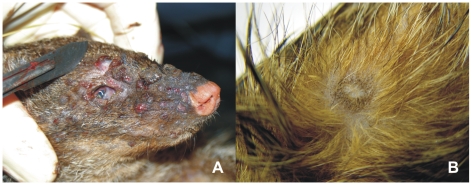
Lesions on mongooses #1 and #8. (A) Acute lesions on the head of mongoose #1 with a generalized infection and (B) subacute to chronic epidermal lesions with scarring on the body of mongoose #8.

The affected colony of banded mongooses comprised 6 males and 7 females housed together for more than 6 months (Table 2). On January 28^th^ 2008 a juvenile female mongoose (#1) was found dead with a large number of ulcerated skin lesions distributed mainly on head, extremities and genitals (Fig. 1, A). Although all the other animals appeared to be clinically healthy at that time, the whole group was treated with an oral antibiotic (Amoxicillin) over their food. The arrangement of the enclosure with a variety of animal-made holes and burrows made individual trapping and examination of animals impossible. On February 1st a juvenile male (#2) was found dead with similar clinical signs. Four days later two symptomatic adult animals (male #3 and female #4) with reduced motility and dermal lesions were trapped and euthanized. By that time the causative agent had been identified as CPXV and quarantine measures were put into place to prevent further spread to other animals. Access was restricted to two animal keepers and the veterinarian, all of whom had to use stringent disinfection measures. Live traps were installed to catch all remaining mongooses. Of the eight mongooses trapped and euthanized on February 12, three animals displayed macroscopically visible, randomly distributed subacute to chronic epidermal lesions with scarring as well as some sparsely haired foci (Fig. 1, B). The other five animals were clinically and histopathologically unremarkable. After the last animal was trapped on February 15 also with dermal lesions, the pen was immediately covered with quick lime and the soil in the pen subsequently removed.

**Table 2:** Clinical and laboratory findings in a cowpox virus outbreak affecting a colony of 13 banded mongooses (*Mungos mungo*) and 2 jaguarundis (*Herppailurus yagouaroundi*) at Krefeld Zoo, Germany. Skin, lung, liver, tongue, spleen and feces were tested by real-time PCR; blood by IFAT.

Animal	Sex	Age in years	Case history	Lesions (Frequency)	Lesions (Location)	Skin	Lung	Liver	Tongue	Spleen	Feces	Blood
M #1	F	<1	Died, 28 Jan 2008	Multiple	Skin^1^, lung, liver	+	n.d. 8	n.d. 8	n.d.	n.d.	n.d.	n.d.
M #2	M	<1	Died, 01 Feb 2008	Multiple	Skin^2^, lung, liver	+	+	−	+	−	−	n.d.
M #3	M	4	Euthanized, 05 Feb 2008	Multiple	Skin^3^, lung, liver	n.d.	n.d.	n.d.	n.d.	n.d.	n.d.	n.d.
M #4	F	5	Euthanized, 05 Feb 2008	Multiple	Skin, lung, liver	n.d.	n.d.	n.d.	n.d.	n.d.	n.d.	n.d.
M #5	M	<1	Euthanized, 12 Feb 2008	No lesions		−	−	−	−	−	−	10,000^5^
M #6	M	4	Euthanized, 12 Feb 2008	No lesions		+	−	−	−	−	−	10,000^5^
M #7	F	5	Euthanized, 12 Feb 2008	No lesions		−	n.d.	−	+	−	−	1,000^5^
M #8	F	4	Euthanized, 12 Feb 2008	Sparse	Skin (scars)	+	−	−	+	−	−	10,000^5^
M #9	F	<1	Euthanized, 12 Feb 2008	No lesions		+	−	−	−	−	−	1,000^5^
M #10	M	4	Euthanized, 12 Feb 2008	Sparse	Scrotum (scars)	+	+	−	–	–	–	10,000^5^
M #11	F	4	Euthanized, 12 Feb 2008	No lesions		–	n.d.	–	–	–	–	10,000^5^
M #12	M	<1	Euthanized, 12 Feb 2008	Sparse	Skin (scars)	–	–	–	–	+	–	10,000^5^
M #13	F	4	Euthanized, 15 Feb 2008	Multiple	Skin (scars)	+	–	–	+	–	–	10,000^5^
J #1	F	<1	First signs of illness, 19 Feb 2008; started to recover, 26 Feb 2008	Multiple	Skin^4^	+^6^	n.d.	n.d.	n.d.	n.d.	n.d.	20,480^6^
J #2^7^	M	1	First signs of illness, 26 Feb 2008	Sparse	Skin							100^6^
			Died, 06 Mar 2008	Sparse	Skin, larynx	+	+	+	+	+	+	10,000^5^

Positive virus isolates:

1 CPXV MonKre08/1,

2 CPXV MonKre08/2,

3 CPXV MonKre08/3,

4 CPXV JagKre08/1,

5 Sera were collected on the day of death,

6 Sera and skin scrapings collected on 20 Feb 2008,

7 J#2 also PCR positive for lymph nodes, larynx and colon,

8 Poxvirus infection verified by histology,

M: mongoose, J: jaguarondi.

IFAT: Indirect fluorescence antibody test detecting specific anti-orthopoxvirus antibodies with protein G (mongoose) or α-feline (jaguarundi) as secondary antibodies, the reciprocal titer is given.

**Figure 2:**
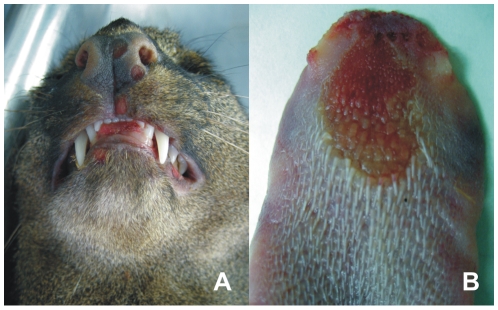
Acute lesions of jaguarundi #2. (A) Round, “punched-out” erosions at the mucosal surface of nose and lips and (B) typical lesions at the dorsal aspect of the tongue.

Despite quarantine precautions, the infection spread to a female jaguarundi in which an unusual behavior of frequent scratching was observed on February 19. The female jaguarundi and a male jaguarondi were housed in another building separated by a main path from the mongoose enclosure. Both jaguarundis were cared for by keepers who also looked after the mongooses and had never showed any clinical signs of CPXV infection. Both jaguarundis were immobilized on February 20 for the collection of sera and further examination. While the male cat presented without any clinical findings and revealed a low orthopoxvirus-specific antibody titer of 1∶100, the female animal had several small ulcerated skin lesions distributed all over its body and an antibody titer of 1: 20,480. Both were treated with antibiotics (Marbofloxacin) and cared for by other keepers who had not been in contact with other carnivores. The female jaguarundi showed a reduced general condition but started to recover after February 26, with visible bald patches on the body. Simultaneously, the male cat's condition deteriorated. It became apathetic and lethargic and died on March 6. At the time of death the animal had an anti-orthopoxvirus (OPV) titer of 1∶10.000. Numerous round, “punched-out” erosions were discovered at the mucosal surface of nose (Fig. 2, A), lips and oral cavity. Particularly the dorsal aspect of the tongue (Fig. 2, B) and larynx were affected, the latter revealed broad confluent ulcers with ridge-like rims of necrotic debris. Similar lesions were sparsely found on the skin of the body.

### Histological and virological investigations

**Figure 3:**
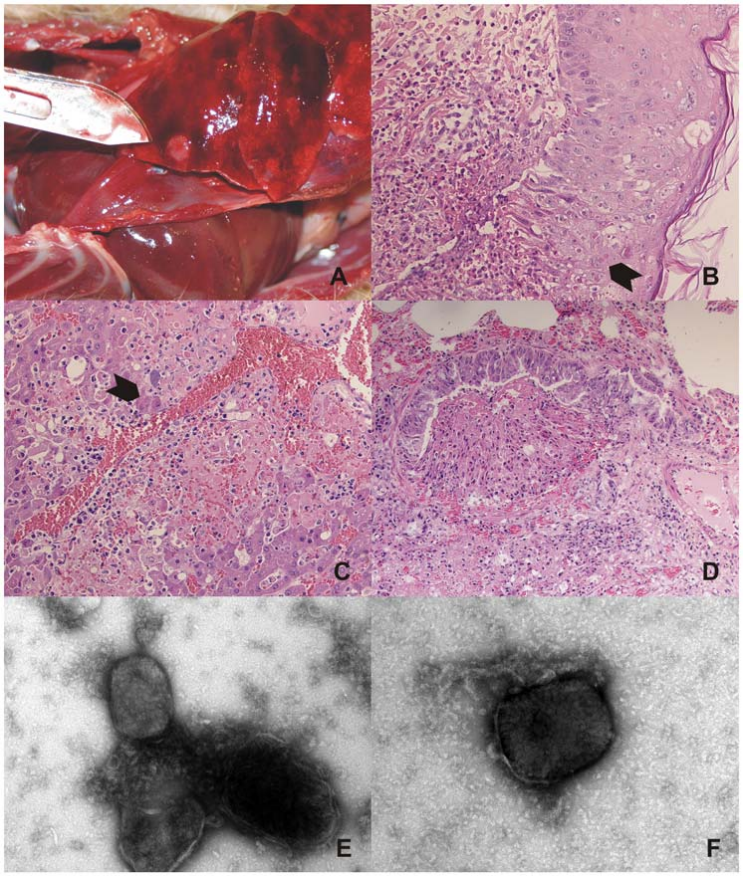
Histopathological and electron microscopical examination. (A) Multiple circumscribed, elevated, pale red, plaque-like foci in the lung of mongoose #1, (B) HE-stained skin lesion of mongoose #1 showing multiple eosinophilic intracytoplasmic inclusion bodies (arrows) and mild ballooning degeneration of epidermal cells associated with focal severe necrotizing dermatitis with neutrophilic and lymphoplasmacellular infiltrates, (C) HE-stained liver section of mongoose #1 showing severe necrosis with hemorrhage and mild inflammatory infiltration and degenerating hepatocytes with multiple intracytoplasmic inclusion bodies (arrow), (D) HE-stained lung section of mongoose #1 showing a bronchiolus with markedly hyperplastic epithelium and focal obliterating proliferation undergoing necrosis. Negative-stain electron microscopy revealing typical orthopoxvirus-like particles in skin lesion material of mongoose #1 (E) and jaguarundi #1 (F).

The first four mongooses (#1–4) revealed large numbers of ulcerated skin lesions distributed over the body (Fig. 1, A) and bacterial superinfections caused by various bacterial species. Their lungs and livers displayed multiple circumscribed, elevated, pale red, plaque-like foci of up to 1 cm in diameter (Fig. 3, A). Poxvirus infection was provisionally diagnosed when skin sections of the first mongoose revealed multifocal intracytoplasmatic eosinophilic inclusion bodies especially characteristic of cowpox virus (Fig. 3, B). Interestingly, the histological equivalent of the macroscopically visible foci in liver and lung were acute to subacute focal necroses with numerous intralesional eosinophilic intracytoplasmatic inclusion bodies in epithelial cells (Fig. 3, C,D). In addition, extensive hyperplasia of the epithelium of bronchioles and multifocal intravascular accumulations of bacteria were found in these foci. Poxvirus infection was confirmed by negative-stain electron microscopy revealing typical orthopoxvirus-like particles in skin lesion material from both mongooses and jaguarundi (Fig. 3, E,F). The morphological features of hemorrhagic pocks on the chorioallantoic membrane (CAM) of infected embryonated hen's eggs indicated CPXV. Virus was isolated post-mortem from skin samples of three different animals (CPXV MonKre08/1, 08/2, 08/3). PCR analysis and sequencing of the complete hemagglutinin (HA) open reading frame (ORF) identified the causative agent to be CPXV. By the time these results became available, the first four animals had died and attempts to capture the remaining animals in live traps were initiated.

Additional analysis of organ specimens from the remaining animals (# 5–#13) by quantitative real-time PCR revealed CPXV DNA in several organs of all but two mongooses, indicating infection in three out of five mongooses which were histopathologically unremarkable (Table 2). Finally, infection of all mongooses was confirmed by the detection of high titers of orthopoxvirus-specific antibodies in blood samples taken post-mortem by an indirect fluorescent antibody test (IFAT). The animals without clinical signs or positive PCR results also revealed high titers, indicating recent infection.

A CPXV strain was also isolated from a skin lesion of the female jaguarundi (CPXV JagKre08/1). Although the female jaguarundi showed clinical signs and revealed a very high orthopoxvirus-specific antibody titer at the beginning of the CPXV infection, she was able to recover. In contrast, the male jaguarundi which revealed only a low antibody titer at the same sampling time died after 15 days. Interestingly, all 9 organ samples of the male cat contained large amounts of CPXV DNA. In comparison to all other animal organs investigated, additional specimens from the male jaguarundi were found to be positive, for example, feces, lymph nodes, larynx and colon.

### Human CPXV infections

**Figure 4:**
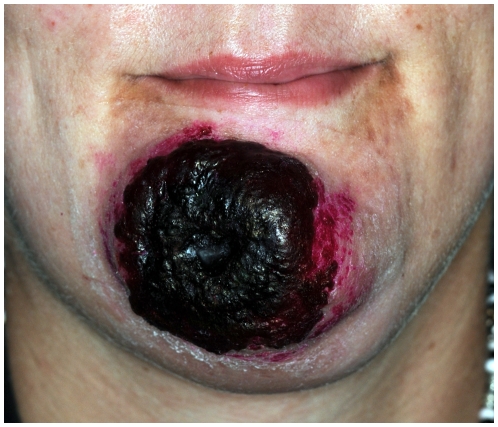
Severe cowpox lesion on the patient's chin caused by the identical virus strain that was isolated from deceased mongooses and jaguarundis.

During early spring of 2008 four human CPXV infections occurred in the city of Krefeld and in the surrounding area (not published). Virus was isolated from all human cases and due to their sequence identity was named CPXV HumKre08/1. The source of infection was traced to CPXV-infected pet rats which all had died after purchase from local pet shops. In September 2008 another case of CPXV infection was diagnosed in an employee (Fig. 4) of a private reptile zoo in Landau, more than 300 km away from Krefeld (not published). Virus was isolated from the patient's chin and was named CPXV HumLan08/1. The HA-gene sequence of both virus strains (Table 3) proved to be different (see below).

**Table 3:** Characteristics of cowpox virus isolates and orthopoxvirus reference strains.

Orthopoxvirus strain	Host	Location	Year	Size of the ORF of the hemagglutinin gene	Accession number of the hemagglutinin gene/reference
CPXV HumLan08/1	Human	Landau/GE	2008	921 bp	GQ260460
CPXV JagKre08/1	Jaguarundi	Krefeld/GE	2008	921 bp	GQ260459
CPXV MonKre08/1	Mongoose	Krefeld/GE	2008	921 bp	GQ260457
CPXV MonKre08/2	Mongoose	Krefeld/GE	2008	921 bp	GQ281042
CPXV MonKre08/3	Mongoose	Krefeld/GE	2008	921 bp	GQ260458
CPXV HumKre08/1	Human	Krefeld/GE	2008	924 bp	GQ260461
CPXV EleGri07/1	Elephant	Grimmen/GE	2007	921 bp	[8]
CPXV HumGri07/1	Human	Grimmen/GE	2007	921 bp	[8]
CPXV RatGri07/1	Rat	Grimmen/GE	2007	921 bp	[8]
CPXV Brighton Red	Human	Brighton/UK	1937	894 bp	NC_003663
CPXV Catpox 5	Cheetah	London/UK	1982	894 bp	AY902263
CPXV HumNL02/1	Human	Utrecht/NL	2002	942 bp	[36]
CPXV Rat Moscow	Rat	Moscow/RU	1973	942 bp	AY902263
CPXV RatNL03/1	Rat	Almere/NL	2003	942 bp	[14]
CPXV CatHan04/1	Cat	Hannover/GE	2004	939 bp	[37]
CPXV Biber V940/97	Beaver	Berlin/GE	1997	936 bp	[18], AY902260
CPXV Katzenbaer	Red Panda	Berlin/GE	1997	936 bp	[18], AY902261
CPXV GRI-90	Human	Moscow/RU	1990	945 bp	X94355
CPXV OPV 91-3	Human	Munich/GE	1991	951 bp	DQ437593
CPXV Udine	Cat	Udine/Italy	2006	948 bp	EF612709
VACV Copenhagen	nk	nk	nk	948 bp	M35027
VACV Lister	nk	nk	nk	948 bp	AY678276
VACV rabbitpox	Rabbit	Utrecht/NL	nk	939 bp	AY484669
CMLV M-96	Camel	Kazachstan	1996	960 bp	NC_003391
ECTV Moscow	Mouse	Moscow/RU	nk	846 bp	NC_004105
MPXV mpv-utr	Monkey	NL	1965	942 bp	AF375113
VARV Butler	Human	UK	1952	942 bp	AF375129
VARV India	Human	India	1967	957 bp	Y16780

CPXV: cowpox virus, VACV: vaccinia virus, CMLV: camelpox virus, MPXV: monkeypox virus, ECTV: ectromelia virus, VARV: variola virus.

nk: not known.

### Sequencing and phylogenetic analysis

**Figure 5:**
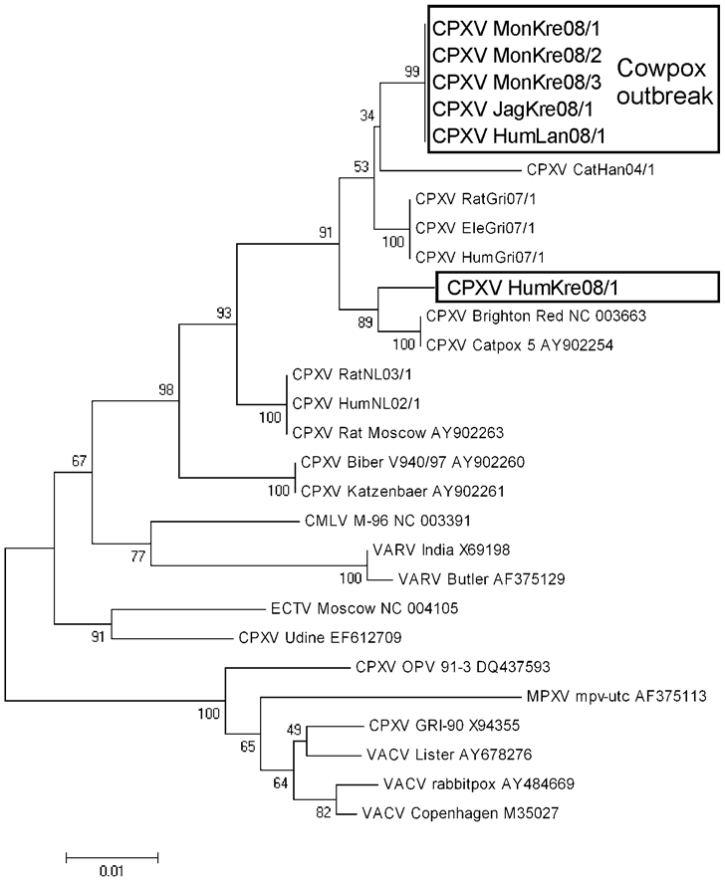
Evolutionary relationships of orthopoxvirus isolates from the outbreak described here and orthopoxvirus reference strains. The evolutionary history was inferred using the Neighbor-Joining method [27]. The optimal tree with the sum of branch length  = 0.28120272 is shown. The percentage of replicate trees in which the associated taxa clustered together in the bootstrap test (500 replicates) is shown next to the branches. The tree is drawn to scale, with branch lengths in the same units as those of the evolutionary distances used to infer the phylogenetic tree. The evolutionary distances were computed using the Maximum Composite Likelihood method and are in the units of the number of base substitutions per site. Codon positions included were 1st+2nd+3rd+Noncoding. All positions containing gaps and missing data were eliminated from the dataset (complete deletion option). There were a total of 753 positions in the final dataset. Phylogenetic analyses were conducted in MEGA4.

The HA-gene sequences obtained from the four CPXV isolates of the outbreak in the Krefeld Zoo (CPXV MonKre08/1, 08/2, -08/3 and CPXV JagKre08/1) were all 921 bp in length and 100% identical to each other (Table 3). BLAST search confirmed the identification as a CPXV with a unique HA-gene sequence not reported so far. Interestingly, this sequence proved to be 100% identical to isolate CPXV HumLan08/1 which was isolated 7 months later and was geographically separated by more than 300 km. Surprisingly, the HA-gene sequence of CPXV HumKre08/1 transmitted from pet rats isolated in the town of Krefeld differed considerably from this cluster: the ORF (924 bp) contains a 3 bp insertion and differs in 25 nucleotides. Accession numbers of all CPXV isolates are indicated in table 3. The results of a phylogenetic analysis (Fig. 5) clearly demonstrate that the two geographically related outbreaks (mongoose and humans in Krefeld) were indeed caused by a different virus but on the other hand the geographically distant cases (human cases in Krefeld and Landau) were caused by the same virus type.

### Vaccination of carnivores

After the death of the male jaguarundi and as a preventative measure to counter the apparently unrestricted transmission of CPXV, all felids and several other carnivores were vaccinated with MVA. To monitor an increase of OPV-specific antibody titers by IFAT, serum samples were collected and tested before and after vaccination (Table 4). Sera collected from the animals at an earlier time were also included. In all but one animal species vaccinated a significant increase in the antibody titer with at least three serial log2 dilutions could be verified, indicating seroconversion. The antibody titer of most animals rose five-fold and more during a four-month period, including cheetah “Otwani”, jaguar “Jackson”, tiger “Sutera”, serval “Nero” and both red pandas “Gorbi” and “Kosima”. Only the otter “Titus” did not show a seroconversion after vaccination. Interestingly, pre-vaccination sera from several animals, for example, snow leopard “Odette” and bush dog “618AC9A”, revealed pre-existing high antibody titers. Also, both animals revealed similar antibody titers in samples taken before the recent CPXV outbreak, indicating the occurrence of a previous infection. The cheetah “Kasai” revealed a titer increase between the retrospective sera and the pre-vaccination sera, indicating a possible infection during the recent outbreak. The female jaguarundi “#1” which had recovered from the recent infection did not further increase its antibody titer significantly after vaccination.

**Table 4:** Orthopoxvirus-specific antibody titers, measured by IFAT, in animals at Zoo Krefeld, Germany, before and after intramuscular vaccination with modified VACV Ankara (MVA).

Animal	Name	Sex	Up to one year before vaccination	2 weeks before vaccination^6^	8–12 weeks after vaccination^7^
			n.d.	80	2,560
	Kasai	M	160^1^	640	n.d.
Jaguar^8^ *(Panthera onca)*	Jackson	M	100^1^	80	2,560
Tiger^8^ *(Panthera tigris)*	Beludru	M	n.d.	160	2,560
	Sutera	F	n.d.	320	20,480
Snow leopard^8^ (*Uncia uncia*)	Odette	F	1,000^2,3^	1,280	10,240
Serval^8^ (*Lepailurus serval*)	Nero	M	n.d.	80	5,120
	Mutter	F	160^4^	80	n.d.
Jaguarundi^8^ (*Herppailurus yagouaroundi*)	#1	F	n.d.	20,480	40,960
Red Panda^9^ *(Ailurus fulgens)*	Gorbi	M	n.d.	<10	320
	Kosima	F	n.d.	<10	640
Bush Dog^10^ *(Speothos venaticus)*	618AC9A	M	2,560^5^	5,120	n.d.
European Otter^11^ *(Lutra lutra)*	Titus	M	n.d.	<10	10

Date of sera sampling: ^1^24 May 2007, ^2^12 Dec 2007, ^3^30 Jan 2008, ^4^29 Jun 2007, ^5^14 Mar 2007.

Date of first vaccination: ^6^03/05–25/08.

Date of second vaccination: ^7^11 Apr 2008–7 May 2008.

Secondary antibodies: ^8^α-feline, ^9^Protein A/G, ^10^α-canine, ^11^Protein A.

IFAT: Indirect fluorescence antibody test detecting specific anti-orthopoxvirus antibodies, the reciprocal titer is given.

n.d.  =  not done.

Other non-carnivorous animal species housed in the same area of the zoo were also tested for orthopoxvirus-specific antibodies, including camel *(Camelus bactrianus)*, tapir *(Tapirus terrestris)*, kudu *(Tragelaphus strepsicornis)*, duiker *(Cephalophus monticola)*, muskox *(Ovibos moschatus)*, springbok *(Antidorcas marsupialis)* and barashinga *(Cervus duvauceli)*. None of these animals revealed significant antibody titers (data not shown).

## Discussion

CPXV infections are increasing in Germany despite being described as an extremely rare zoonotic infection. During the two years before the current outbreak (2006/07), 22 human cases have been diagnosed, in addition to numerous cases in pet cats and exotic zoo animals including elephants. While it cannot be excluded that this is due to a reporting bias, this increase may reflect the fact that a smaller proportion of people have immunity against CPXV following the cessation of smallpox vaccinations. Although the lack of significant evidence for increasing case numbers due to the cessation of smallpox vaccination has been discussed [9], we detected all recent human cowpox cases in people too young to have been vaccinated against smallpox.

Elephants are, by far, the most frequently infected with CPXV. Over 60 cases of elephant infections have been reported from Germany. These days, most elephants are regularly vaccinated with MVA. Hence, only sporadic cases still occur in unvaccinated elephants. The second most commonly infected group are exotic felids with CPXV outbreaks being reported from the UK, continental Europe and Russia. Although felids are highly susceptible to CPXV, very few cases have been reported so far. Exotic zoo animals that are housed in close proximity to other zoo animals and who come into contact with wild rodents and animal keepers are likely to be more susceptible to CPXV infections. Similar epidemics involving animals of different species have been reported only from Moscow in 1973/74, Berlin in 1997 and Almere, the Netherlands, in 2003 [5], [14], [18].

Only occasionally is a definite source of infection identified. Either food rodents in a breeding facility are infected from wild rodents [5] or direct transmission from wild rats occurs [8], [14]. However, the source of infection is often only speculated to be from wild rodents, particularly mice, as they are believed to be the main reservoir for CPXV. For this outbreak with two separate cases in geographically distant areas infected with the same CPXV strain, transmission via wild rodents as the initial source could be ruled out. Other transmission pathways, e.g. contact with infected cats, exchange of exotic animals, keepers or rodents from their own breeding facility, could also be ruled out. Nevertheless, like most carnivores at Krefeld Zoo, mongooses and jaguarundis were regularly fed with thawed rats (in addition to other meat) by the same two keepers, suggesting that rats purchased from an animal food supplier were the most probable source of infection.

The appearance of well-developed clinical symptoms in the first four mongooses during a seven-day period suggests a single common source of infection about 1–2 weeks earlier from the same food source. The remaining nine mongooses revealed high levels of antibodies and healed skin lesions about two weeks after the first mongoose had died, indicating a similar time course of infection as the four previous cases and also that no interspecies transmission among mongooses had occurred. Transmission of the identical CPXV strain to the female jaguarundi occurred at a later time as indicated by the delayed appearance of clinical symptoms. Since no direct contact between mongooses and jaguarundis was possible, an indirect and accidental transmission by one of their keepers seems to be the most probable source of infection despite the applied safety measures. The fact that the appearance of clinical signs in the male jaguarundi was delayed by one week suggests an interspecies transmission by direct or indirect contact with contaminated saliva, urine or feces.

In contrast to previous reports where hemorrhagic pneumonia was the main post-mortem finding in infected felids, in both the mongooses and the jaguarundis a prolonged, exclusively dermal type of CPXV infection was observed with lesions distributed over the whole body. Among the group of mongooses the first four infected animals displayed an acute systemic CPXV infection, whereas the latter nine animals were already in the process of healing. The mortality rate was about 30% and no correlation between disease outcome and age or sex could be found. High antibody titers detected in serological analyses indicated a 100% susceptibility of CPXV for mongooses and jaguarundis. Although housed in separate buildings and in different areas of the zoo, several other felids and carnivores including cheetah “Kasai”, snow leopard “Odette” and the bush dog also revealed high antibody titers, pointing towards a high CPXV infection rate during the recent outbreak or previous exposure to CPXV. In other extensive studies no evidence of CPXV antibodies was found in 93 captive exotic animals including cheetahs, lions and tigers [19] as well as in an ongoing serological survey of cats in British zoos involving over 100 cats [20]. Despite the high seroprevalence verified in numerous carnivores in Krefeld and also in sera tested retrospectively, a CPXV outbreak had never been suspected previously which indicates a high susceptibility rate to the circulating virus strain(s) but a low mortality rate among felids.

As recognized in the female jaguarundi, a significant orthopoxvirus-specific antibody titer early during a CPXV infection seems crucial for survival. This case highlights the need for further extended vaccination studies leading to increased efforts toward the general vaccination of potentially susceptible and rare exotic animals. In contrast to previous reports about the absence of an immunological response after being vaccinated with a smallpox vaccine [19], the routinely performed vaccination of elephants with MVA seems to induce a prolonged immune response and protect the immunized animals from a symptomatic CPXV infection as there have not been any reports of vaccinated elephants becoming infected by CPXV. In this study a significant increase of the antibody titer was achieved in all vaccinated felids (including cheetah, jaguar, tiger, snow leopard and serval) but also in red pandas which were previously reported as being susceptible to CPXV in two fatal cases [18]. In addition to several canid species that have already been reported to be susceptible to CPXV infection including red fox and domestic dogs [10], [21], bush dogs were also proven to be susceptible as they showed unexpectedly high antibody titers.

The time-delayed appearance of the clinical signs in both jaguarundis followed by the death of the animal infected later indicated one of the management problems during a CPXV outbreak. When animals known to be or suspected to be susceptible to CPXV reveal typical signs of an infection, they should immediately be separated from other animals by applying strict quarantine measures, treated with antibiotics against secondary bacterial infections and observed closely for at least three weeks. Nevertheless, prompt segregation of potentially infected animals may not be possible due to lack of separate pens available at the crucial time. Further, it is impossible to permanently segregate zoo animals from wild rodents. A continuous control of food animals might be hard or impossible to accomplish, especially when purchased from different wholesale dealers or animal husbandries. Since no effective and approved treatment for animals in case of CPXV infections is available, e.g. previous trials with γ-globulin were not successful [19] and the new therapeutic compound ST-246 is not approved yet [22], only prophylactic vaccination might protect zoo animals. One major difficulty is to validate the protective effect after a vaccine take in different species. Zoo animals are usually too scarce and valuable to permit trials with the potentially effective MVA vaccine and a controlled challenge with CPXV. More effort is needed to monitor the success of MVA vaccinations, since a positive effect has already been recognized in elephants. As shown here, positive immunological boosts could be demonstrated in most felids and red pandas but not in otters.

Our study has revealed that two species (*Mungos mungo* and *Herpailurus yagouaroundi*) are susceptible to CPXV infection and this was unknown previously. We also observed high orthopoxvirus-specific antibody titers in unvaccinated zoo animals that did not show symptoms of infection, suggesting that there are a far higher number of CPXV infections than is generally hypothesized. The probable existence of additional unknown CPXV hosts may present a risk to other exotic animals but also to the general public, as was shown in this outbreak. With the cessation of the smallpox vaccination, younger humans are susceptible to CPXV infection and we expect to see an increased incidence of infection in humans in the future.

## Methods

### Specimen preparation

All samples were taken from animals after immobilization or euthanization and none of the animals examined had previously been vaccinated. Serum samples were taken before and after vaccination with modified vaccinia virus Ankara (MVA) or had been taken previously during routine examinations. All samples were kept frozen at −20°C until further use. For the human patient, for diagnostic evaluation a swap sample was taken directly from the lesion and processed further for routine PCR and sequence analysis [23]. No specific ethical approval was needed since the human sample was taken for diagnostic purpose and the results were obtained from routine diagnostic analyses.

### Histology

Tissue samples were fixed in 10% buffered formalin, processed routinely, embedded in liquid paraffin and sectioned at 3 µm. Slides were stained with hematoxylin and eosin (HE).

### Electron microscopy

Homogenized skin lesions were centrifuged at low speed to remove debris and processed for negative staining electron microscopy as described elsewhere [24]. Briefly, 400-mesh copper grids covered with pioloform F and carbon were floated on sample drops, washed twice on drops of double-distilled water and contrasted with 1% uranyl acetate (60 mM, pH 4). Prepared grids were then examined by transmission electron microscopy under an FEI Tecnai G2 electron microscope

### Indirect fluorescent antibody test

The titer of orthopoxvirus-specific antibodies in animal sera were determined by immunofluorescence staining of CPXV-infected human cells that was performed according to standard procedures [25]. Briefly, CPXV-infected HEp2 cells (MOI 0.1) were grown on glass slides for 24 h at 37°C. Cells were fixed in 4% formalin and incubated with serial dilutions of the animal serum, followed by a FITC-conjugated secondary antibody or protein A/G, depending on the animal species (see Tables 2 and 4), counterstained with Evans Blue and evaluated by fluorescence microscopy.

### Real-time PCR and sequencing

DNA from skin lesions and other organs was prepared using a Qiagen Blood kit according to the manufacturer's instructions (Qiagen, Hilden, Germany). Quantitative real-time PCR amplification was applied to detect orthopoxvirus DNA [26]. To obtain species identity of virus isolates, the products of a PCR spanning the entire open reading frame (ORF) of the hemagglutinin (HA) gene [23] were sequenced. Data sets were edited and aligned using BioEdit.

### Phylogenetic analysis

Phylogenetic analysis of sequences of the entire open reading frame (ORF) of the HA gene of virus isolates described here and orthopoxvirus reference strains (Table 3) were performed with the MEGA 4.0 software suite (www.megasoftware.net) using the neighbor-joining method [27].

### Animal vaccination

As a protective measure against CPXV infections various zoo animals were vaccinated twice intramuscularly with an interval of 5 weeks by blowpipe with 2 ml of modified vaccinia virus Ankara (MVA) obtained from the University of Munich, Institute for Medical Microbiology, Infectious and Epidemic Diseases, Germany (Dr. Werner Eichhorn).
